# Hierarchical deep learning of multiscale differential equation time-steppers

**DOI:** 10.1098/rsta.2021.0200

**Published:** 2022-08-08

**Authors:** Yuying Liu, J. Nathan Kutz, Steven L. Brunton

**Affiliations:** ^1^ Department of Applied Mathematics, University of Washington, Seattle, WA 98105, USA; ^2^ Department of Mechanical Engineering, University of Washington, Seattle, WA 98105, USA

**Keywords:** deep learning, multiscale modelling, model discovery, scientific computing, dynamical systems

## Abstract

Nonlinear differential equations rarely admit closed-form solutions, thus requiring numerical time-stepping algorithms to approximate solutions. Further, many systems characterized by multiscale physics exhibit dynamics over a vast range of timescales, making numerical integration expensive. In this work, we develop a hierarchy of deep neural network time-steppers to approximate the dynamical system flow map over a range of time-scales. The model is purely data-driven, enabling accurate and efficient numerical integration and forecasting. Similar ideas can be used to couple neural network-based models with classical numerical time-steppers. Our hierarchical time-stepping scheme provides advantages over current time-stepping algorithms, including (i) capturing a range of timescales, (ii) improved accuracy in comparison with leading neural network architectures, (iii) efficiency in long-time forecasting due to explicit training of slow time-scale dynamics, and (iv) a flexible framework that is parallelizable and may be integrated with standard numerical time-stepping algorithms. The method is demonstrated on numerous nonlinear dynamical systems, including the Van der Pol oscillator, the Lorenz system, the Kuramoto–Sivashinsky equation, and fluid flow pass a cylinder; audio and video signals are also explored. On the sequence generation examples, we benchmark our algorithm against state-of-the-art methods, such as LSTM, reservoir computing and clockwork RNN.

This article is part of the theme issue ‘Data-driven prediction in dynamical systems’.

## 1. Introduction

Scientific computing has revolutionized nearly every scientific discipline, allowing for the ability to model, simulate, engineer and optimize a complex system’s design and performance. This capability has been especially important in nonlinear, multiscale systems where recourse to analytic and perturbation methods are limited. For instance, modern high-fidelity simulations enable researchers to design aircraft, simulate the evolution of galaxies, quantify atmospheric and ocean interactions for weather forecasting, and model high-dimensional neuronal networks of the brain. Thus, given a set of governing equations, typically spatio-temporal *partial differential equations* (PDEs), discretization in time and space form the foundational algorithmic structure of scientific computing [1–3]. Discretization is required to accurately resolve all relevant spatial and temporal scales in order to produce a high-fidelity representation of the dynamics. Such resolution can be prohibitively expensive, as resolving physics on fast time scales limits simulation times and the ability to model slow timescale processes [4,5]. Time-stepping schemes are typically based on Taylor series expansions, which are local in time and have a numerical accuracy determined by the step size Δt. However, there is a growing effort to develop *deep neural networks* (DNNs) to learn time-stepping schemes unrestricted by local Taylor series constraints [6–9]. We build on the flow map viewpoint of dynamical systems [8,10,11] in order to learn *hierarchical time-steppers* (HiTSs) that explicitly exploit the multiscale flow map structure of a dynamical system over a disparate range of time-scales. In leveraging features at different timescales, we can produce an accurate and efficient computational scheme that can provide exceptional efficiency in long-time simulation/forecasting and that can be integrated with classical time-stepping algorithms.

Numerical discretization has been extensively studied since the earliest days of scientific computing. Numerical analysis has provided rigorous estimates of error bounds for the diversity of discretization schemes developed over the past few decades [1–3]. Spatial discretization predominantly involves finite element, finite difference or spectral methods. Multigrid methods have been extensively developed in physics-based simulations where coarse-grained models must be progressively refined in order to achieve a required numerical precision while remaining tractable [12,13]. The resulting discretized dynamics may be generically represented as a nonlinear dynamical system of the form

1.1 $$\frac{d}{dt}x(t)=f(x(t),t),$$

in terms of a state $x\in R^{D}$ (typically D≫1). The dynamics are then integrated with a time-stepping algorithm. As with spatial discretization, there is a wide range of techniques developed for time-stepping, including explicit and implicit schemes, which have varying degrees of stability and accuracy. These schemes approximate the discrete-time flow map [14,15]

1.2 $$x(t+\Delta t)=F(x(t),\Delta t)≜\int_{t}^{t+\Delta t}f(x(\tau ),\tau )\;d\tau ,$$

often through a Taylor-series expansion. Runge–Kutta, for which the Euler method is a subset, is one of the standard time-stepping schemes used in practice. Generically, it takes the form

1.3 $$x_{n+1}\approx {\tilde{F}}_{\Delta t}(x_{n})≜x_{n}+\Delta t\sum_{j=1}^{k}b_{j}h_{j},$$

where

1.4 $$h_{j}=f\left(x_{n}+g\left(\sum_{k=1}^{j-1}\alpha_{j,k}h_{k}\right),t_{n}+c_{j}\Delta t\right)$$

and $x_{n}=x(t_{n})=x(n\Delta t)$. Note that the contributions $h_{j}$ are hierarchically computed in the Runge–Kutta scheme. The weightings $b_{j}$, $c_{j}$ and $\alpha_{j,k}$ are derived from Taylor series expansions in order to minimize error. For instance, the classic fourth-order Runge–Kutta scheme, for which k=4 above, has a local truncation error of $O(\Delta t^{5})$, which leads to a global time-stepping error of $O(\Delta t^{4})$. Euler stepping, for which k=1, has local and global time-stepping errors of O(Δt) and $O(\Delta t^{2})$, respectively. Importantly, the error is explicitly related to the time-step Δt, making such time-discretization schemes *local* in nature.

In contrast to schemes such as Runge–Kutta, that approximate the flow map with a local Taylor series, it is possible to directly construct an approximate flow map $\hat{F}$ using DNN architectures. There are several approaches to modelling flow-map time-steppers using neural networks, which will be reviewed below. The approach taken in this work is to develop a hierarchy of approximate flow maps ${\hat{F}}_{j}(x,\Delta t_{j})$ to facilitate the accurate and efficient simulation of multiscale systems over a range of time-scales; similar flow map composition schemes have been demonstrated to be highly effective for simulating differential equations without neural networks [10,11,16]. Flow maps also provide a robust framework for model discovery of multiscale physics [17,18]. Various existing DNN architectures can be integrated into this hierarchical framework. Flow map approximations based on the Taylor series are typically only valid for small time steps, as the flow map becomes arbitrarily complex for large time steps in chaotic systems. However, DNN architectures are not limited by this small time step constraint, as they may be sufficiently descriptive to approximate exceedingly complex flow map functions [19].

Neural networks have been used to model dynamical systems for decades [20,21]. They are computational models that are composed of multiple layers and are used to learn representations of data [22,23]. Owing to their remarkable performance on many data-driven tasks [24–29], various new architectures that favour interpretability and promote physical insight have been recently proposed, leading to many successful applications. In particular, it has been shown that neural networks may be used in conjunction with classical methods in numerical analysis to obtain discrete time-steppers [6–8,30–33]. Other applications include reduced order modelling [34,35], multiscale modelling [36–39], scientific computing [40–43], coordinate transformations [44,45], attractor reconstructions [46,47], operator learning [48,49] and forecasting [50–53]. Neural network models are increasingly popular for two reasons. First, the universal approximation theorem guarantees that arbitrary continuous functions can be approximated by neural networks with sufficiently many hidden units [54]. Second, neural networks themselves can be viewed as discretizations of continuous dynamical systems [55–61], which makes them suitable for studying dynamics. Among all architectures, *recurrent neural networks* (RNNs) are natural candidates for temporal sequence modelling; however, training has proven to be especially difficult due to the notorious exploding/vanishing gradient problem [62,63]. To alleviate this problem, new architectures have been proposed [64–66], for example, augmenting the network with explicit memory using gating mechanism, resulting in the *long short term memory* (LSTM) algorithm [64], or adding skipped connections to the network, leading to *residual networks* (ResNet) [67].

**Figure 1.**
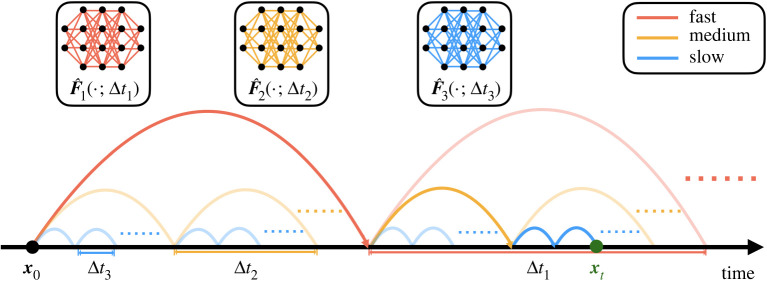
Multiscale hierarchical time-stepping scheme. Here, we employ neural network time-steppers over three time scales. The red model takes large steps, leaving the finer time-stepping to the yellow and blue models. The dark path shows the sequence of maps from $x_{0}$ to $x_{t}$.

In this work, we expand on [8] and employ a deep *residual network* (ResNet) as the basic building block for modelling the flow-map dynamics (1.2). Unconstrained by the typical form of a Taylor-series based time-stepping scheme (1.3), a multiscale modelling perspective is taken to strengthen the performance of our proposed multiscale, flow-map models. Our multiscale HiTS algorithm consists of ResNet models trained to perform hierarchical time-stepping tasks. The contributions of this work are summarized as follows:

— We propose a novel method to couple neural network HiTSs trained across different time scales, shown in figure 1, resulting in more accurate future state forecasts without losing computational efficiency.
— Neural network HiTSs may be coupled with classical numerical time-steppers. This hybrid time-stepping scheme can be naturally parallelized, accelerating classical numerical simulation algorithms.
— By coupling models across different scales, each individual model only needs to be trained over a short period without being exposed to the exploding/vanishing gradient problem, enabling faster training.
— Despite the structural simplicity, the coupled model can still be used to capture long-term dependencies, achieving state-of-the-art performance on sequence generation.

The paper is organized as follows. We motivate the proposed approach and present the methodology in §2. Our approach is then tested on several benchmark problems in §3. In §4, we conclude and discuss future directions. Our code is publicly available at https://github.com/luckystarufo/multiscale_HiTS.

## 2. Multiscale time-stepping with deep learning

**Figure 2.**
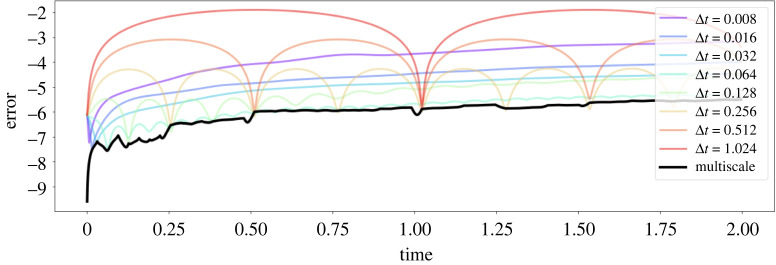
Performance of multiscale HiTS on harmonic oscillator example. This figure shows the time-stepping performance of different neural network time-steppers. One hundred testing trajectories are used for benchmarking each time-stepper and the mean squared errors at each step are plotted in the base-10 logarithmic scale. The black curve represents our proposed multiscale scheme, whereas other colours represent time-steppers at particular scales. (Online version in colour.)

Here we outline our multiscale hierarchical time-stepping based on deep learning, illustrated in figure 1. Our approach constructs a hierarchy of flow maps, ${\hat{F}}_{j}(x,\Delta t_{j})$, each approximated with a deep neural network. This enables accurate and efficient simulations with fine temporal resolution over long time scales, as compared in figure 2. We begin with a motivating example, followed by a summary of notation and description of the training data. Then we will introduce our multiscale hierarchical time-stepping scheme, including descriptions on how to vectorize operations and create hybrid time-steppers by combining with classical numerical methods.

### (a) Motivating example

To explore the effect of time step size on simulation performance, we consider the following simple linear differential equation for a harmonic oscillator

2.1*a* $$\dot{x}=y$$

and

2.1*b* $$\dot{y}=-x.$$

We individually train eight neural networks with step sizes Δt of 0.008, 0.016, 0.032, 0.064, 0.128, 0.256, 0.512 and 1.024 on 500 sampled trajectories and test them on 100 new trajectories. Linear interpolation is used to estimate the state at time steps that are not directly obtained from the neural network time-stepping scheme. To evaluate the forecasting performance, we calculate the averaged error at each time step against the ground truth analytical solution. In this experiment, training and testing data are both sampled from the region $\left\{(x,y)|\;x^{2}+y^{2}\leq 1\right\}$, and we only train one step forward for each neural network time-stepper.

Results are plotted in figure 2, where it is clear that the proposed multiscale time-stepper outperforms all fixed step models. Networks equipped with small time steps offer accurate short-term predictions, although error accumulates at each step and quickly dominates. Networks with large time steps can handle long-term predictions, although they fail to provide information between steps. Time-steppers with some intermediate step sizes (e.g. Δt=0.064) perform well in general as they balance these two factors. However, by leveraging all time steppers across each scale, it is possible to create a multiscale HiTS, given by the black curve, that is both accurate and efficient over long time scales and with fine temporal resolution. Details of the methodology will be presented in the remainder of this section.

### (b) Notation and training data

For training data, we collect n trajectories sampled at p instances with time step Δt:

2.2 $$S^{\left(i\right)}=\{(x_{t}^{\left(i\right)},x_{t+\Delta t}^{\left(i\right)},x_{t+2\Delta t}^{\left(i\right)},\ldots ,x_{t+p\Delta t}^{\left(i\right)})\},$$

for i=0,1,…,n−1. This data is used to train neural network flow map approximations, ${\hat{F}}_{j}(x,\Delta t_{j})$, for j=1,2,…,m.

We follow [8] and employ the residual network as the fundamental building block of our deep learning architecture. Residual networks were first proposed in [67] and have gained considerable prominence since. Specifically, our network only models the difference between time steps $x_{n+1}$ and $x_{n}$, so that it is technically the flow map minus the identity:

2.3 $${\hat{x}}_{t+\Delta t_{j}}=x_{t}+{\hat{F}}_{j}(x,\Delta t_{j}),$$

where

2.4 $${\hat{F}}_{j}(x,\Delta t_{j})=\sigma_{M}({\text{A}}_{M-1}(\cdots \sigma_{1}({\text{A}}_{0})\cdots ))$$

is a feed-forward neural network. The network is parametrized by the linear operators ${\text{A}}_{j}$, and $\sigma_{j}$ are nonlinear activation functions that are chosen to be rectified linear units (ReLU). The extra addition creates a skipped connection from the inputs to the outputs. Here, the architecture ${\hat{F}}_{j}$ learns the increment in states between each $\Delta t_{j}$ step. It is possible to compose the networks to take multiple steps forward in time: ${\hat{F}}_{j}^{\left(k\right)}=\underset{k\;\text{times}}{\underbrace{{\hat{F}}_{j}\circ {\hat{F}}_{j}\circ \cdots \circ {\hat{F}}_{j}}}$. Finally, we formulate our training objective function as

2.5 $$\text{MSE}=\frac{1}{np}\sum_{i=1}^{n}\sum_{k=1}^{p}({\hat{x}}_{t+k\Delta t_{j}}^{\left(i\right)}-x_{t+k\Delta t_{j}}^{\left(i\right)}{)}^{2},$$

which is the classical mean squared loss function.

### (c) Multiscale hierarchical time-stepping scheme

Multiscale modelling is ubiquitous in modern physics-based simulation models. Computational challenges arise in these simulations since coarse-grained macroscale models are usually not accurate enough and microscale models are too expensive to be used in practice [68]. By coupling macroscopic and microscopic models, we hope to take advantage of the simplicity and efficiency of the macroscopic models, as well as the accuracy of the microscopic models [69]. Many efforts have been made towards this goal, resulting in many algorithms that exploit multiscale structure in space and time, including the multi-grid method [70], the fast multipole method [71], adaptive mesh refinement [72], domain decomposition [73], multi-resolution representation [74], multi-resolution dynamic mode decomposition [75], etc. Mathematical algorithms such as heterogeneous multiscale modelling (HMM) [76,77] and the equation-free approach [78] attempt to develop general guidelines and provide principled methods for this field. In this work, however, we develop data-driven models using neural networks, which have different considerations than physics-based simulation models. Regardless, the goal is still to produce accurate and efficient computational models.

Coupling neural network time-steppers across different time scales is rather straightforward, as shown in figure 1. One can clearly see the time-steppers with small Δt are responsible for the accurate time-stepping results over short periods, while the models with larger Δt steps are used to ‘reset’ the predictions over longer periods, preventing error accumulations from the short-time models. Mathematically, suppose we have ${\hat{\text{F}}}_{j}(\cdot ;\Delta t_{j})$ approximating the true flow map of time step size $\Delta t_{j}=2^{m-j}\Delta t\;(j=1,2,\ldots ,m)$ where Δt represents the unit step size. To forecast the state, for example after a time period of $q\Delta t\;(q<2^{m})$, we would need to step forward $q_{j}$ times with model j for j=1,2,…,m, where $q=2^{m-1}q_{1}+2^{m-2}q_{2}+\cdots +2^{0}q_{m}$ is the binary representation of q. All other intermediate time steps will be obtained via linear interpolation. So instead of stepping forward in a sequential manner (i.e. using the smallest scaled model to step forward for q steps), we have reduced the total number of steps to order $O(\log_{2}q)$. There are additional benefits of this multiscale coupling in time. First, training each individual network is simpler, as it is possible to use trajectories with small p so that each model may focus on its own range of interest, circumventing the problem of exploding/vanishing gradients. Second, the framework is flexible, so that for forecasting it is possible to vectorize the computations or utilize parallel computing technologies, enabling fast time-stepping schemes. Moreover, it can be easily combined with classical numerical time-steppers, resulting in hybrid schemes, boosting the performance of simulation algorithms.

The proposed multiscale coupling procedure may resemble those used in multiscale simulations (e.g. HMM). However, the data-driven microscopic cannot provide accurate long-time forecasts on its own, so the coupling here is not only for efficiency but also to improve the long-time fidelity of the model. It should also be noted that before coupling neural network time-steppers across different time scales, cross validation is used to filter the models. This is practically helpful because qualities of different models may vary and we want to couple the best set of models. Specifically, suppose we have m neural network models $\left\{{\hat{F}}_{1},{\hat{F}}_{2},\ldots ,{\hat{F}}_{m}\right\}$, ordered by their associated step sizes. We first determine the upper bound index u so that ensembling $\left\{{\hat{F}}_{1},{\hat{F}}_{2},\ldots ,{\hat{F}}_{u}\right\}$ has the best time-stepping performance among $\left\{{\hat{F}}_{1},{\hat{F}}_{2},\ldots ,{\hat{F}}_{k}\right\}$ for all k. Next, we seek a lower bound index l so that $\left\{{\hat{F}}_{l},{\hat{F}}_{l+1},\ldots ,{\hat{F}}_{u}\right\}$ performs best among $\left\{{\hat{F}}_{k},{\hat{F}}_{k+1},\ldots ,{\hat{F}}_{u}\right\}$ for all k.

#### (i) Vectorization


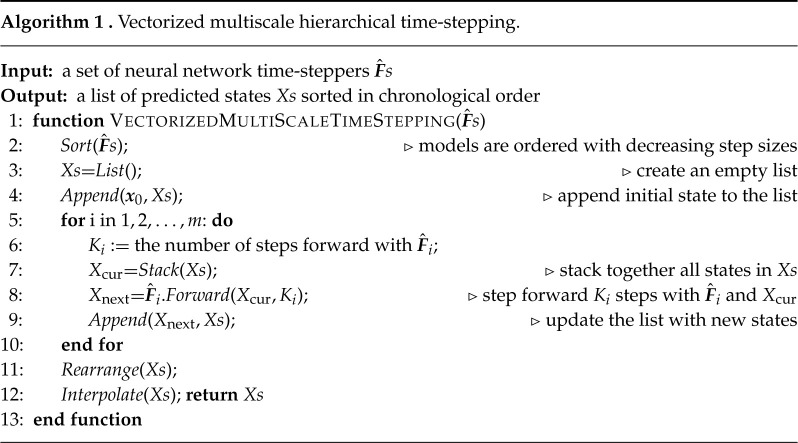


**Figure 3.**
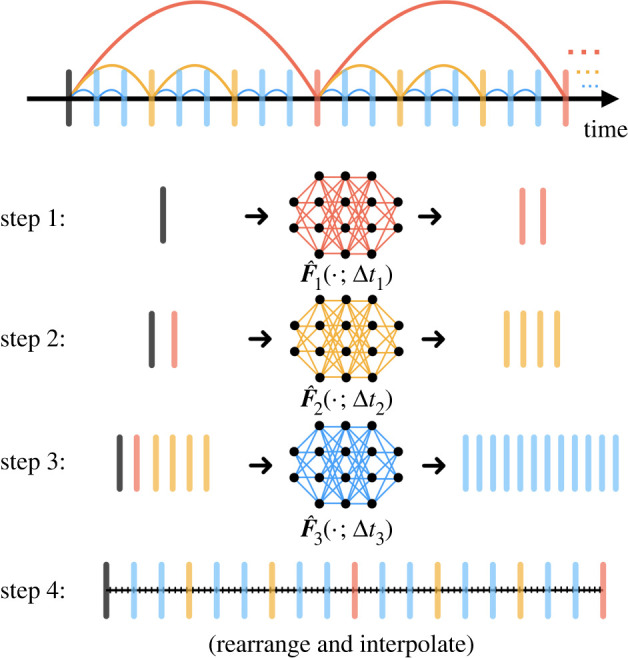
Vectorized computation. The three neural networks in this diagram are used sequentially, ordered by their associated step sizes from large to small. For each network, we stack all currently existing states and step forward (in the beginning, we only use the initial state), resulting in vectorized computations. These newly generated states are further fed to the next neural network in queue. Once we finish using all networks, the states will be rearranged in terms of chronological order and intermediate time steps will be obtained via interpolation. (Online version in colour.)


The diagram for vectorized computations is illustrated in figure 3. The basic idea is to start by using the time-steppers with the largest Δt step and generate the future states corresponding with this stepper. We then stack the new states with the original states and feed them to the next-level neural network time-stepper. After we proceed through all time-steppers, we rearrange the states and use interpolation to fill in the state at all intermediate time steps. Details are given in algorithm 1.

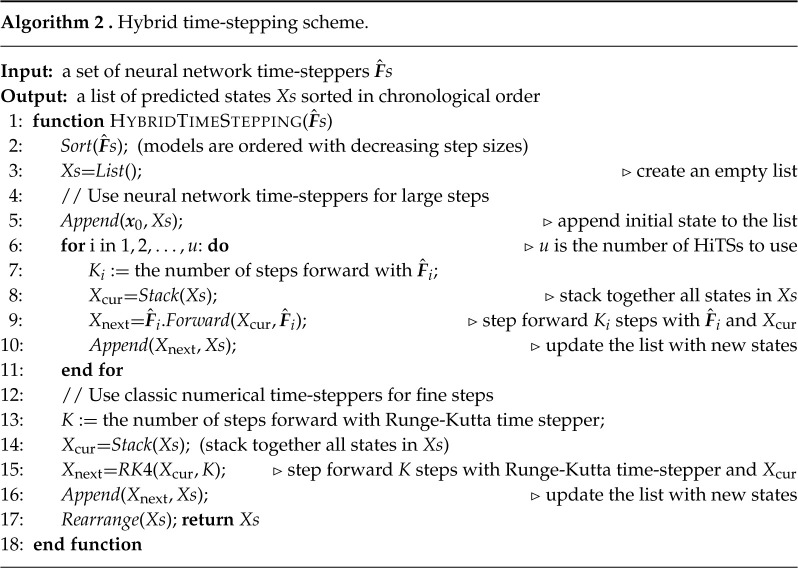


#### (ii) Hybrid time-steppers

**Figure 4.**
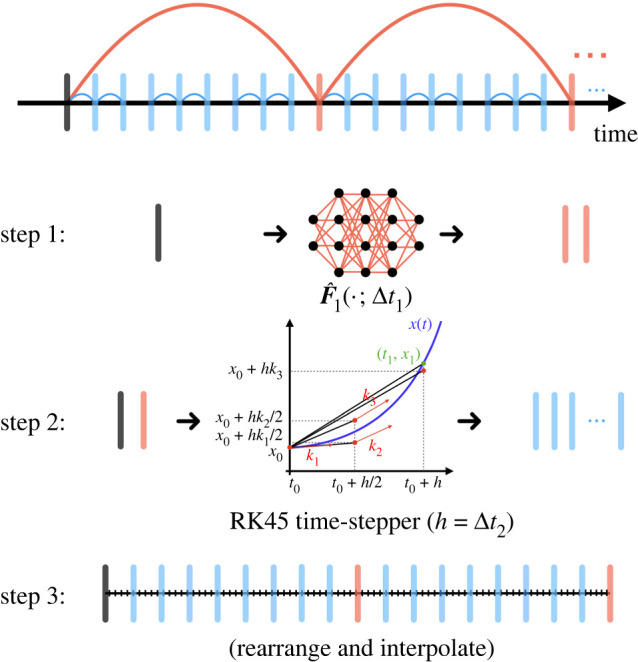
Hybrid time-stepper. A hierarchy of coarse neural network time-steppers generate states that are fed to a fourth order Runge–Kutta solver for fine-scale time-stepping. (Online version in colour.)

The flexibility of our multiscale coupling approach makes it possible to combine these time-steppers with classical numerical time-stepping algorithms. The algorithm is detailed in algorithm 2 and the concept is illustrated in figure 4. This approach provides an innovation in the computational paradigm of numerical simulations. If one were to use classical numerical time-steppers (e.g. Runge-Kutta method) alone, opportunities for vectorized computations or parallel computations are limited due to the serialized nature: one cannot march forward to the future without knowledge about the past. Our hybrid scheme, on the other hand, bypasses the difficulty by using large scaled neural network time-steppers, enabling vectorized computations (or parallel computations) at the bottom level where we use classical numerical time-steppers for accurate simulations.

## 3. Numerical experiments

We will now provide a thorough exploration of our proposed multiscale hierarchical time-steppers. We begin by comparing against single time-scale neural network time-steppers and Runge–Kutta algorithms on simple dynamical systems. We then explore this approach on more sophisticated examples in spatio-temporal physics and sequence generation.

### (a) Benchmark on time-stepping

We first benchmark the multiscale neural network HiTS against the single time-scale neural network time-steppers on five simple nonlinear dynamical systems: a nonlinear system with a hyperbolic fixed point, a damped cubic oscillator, the Van der Pol oscillator, a Hopf normal form and the Lorenz system. For each example, we train 11 single time-scale neural network time-steppers with separate time steps, and then combine them into a multiscale neural network HiTS with the methods described in §c. More details about these numerical experiments can be found in electronic supplementary material, appendix A(a).

Figure 6 shows that the multiscale scheme outperforms all single time-scale schemes in terms of accuracy, as shown by the black curve in the last column. On the sampled testing trajectory, in the third column, our multiscale scheme achieves nearly perfect time-stepping for a time period of 51.20 for the first four nonlinear systems. For the Lorenz system, discrepancy occurs in the forecast after about five time units, when the trajectory switches lobes. Indeed, the error plot suggests the time-stepper becomes unreliable even after a single time unit, due to the intrinsic chaotic dynamics. The challenges of chaos are exacerbated by measurement noise on the data, as shown in the electronic supplementary material, appendix, although there are many applications where low noise make this a viable approach, even for chaotic dynamics. Many works have shown success in predicting the Lorenz system; however, the tasks considered are different from that explored in this work, and they usually fall into the following categories:

— The model is trained with data from a single trajectory and then tested with the same initial state [30,31].
— The resulting model offers accurate short-term predictions while generating similar dynamical statistics for long times [46,79].
— The model predicts the Lorenz system with regular adjustments or intermittent external forcing or under a data assimilation framework [51,80].

By contrast, we train the model with batches of trajectories and test with unseen initial conditions; we also test long-term predictions without any external inputs. In fact, almost all previous models belong to single-scaled models in our discussion.

**Figure 5.**
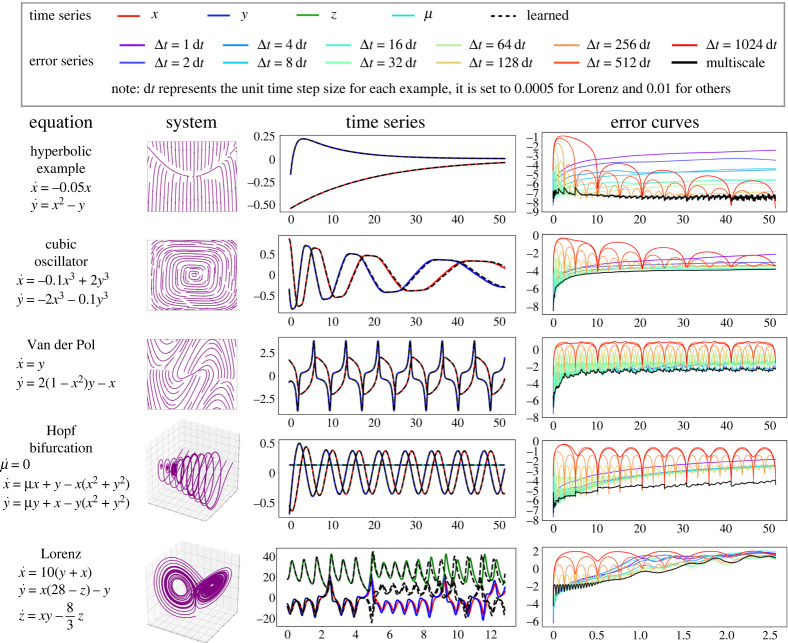
Performance of multiscale HiTSs on nonlinear systems. In the first two columns, systems of equations and phase portraits are visualized. In the third column, we visualize the predictions of multiscale time-steppers on a testing trajectory. In the last column, mean squared errors at each step are visualized in the base-10 logarithmic scale for different time-steppers. (Online version in colour.)

The trade-off between computational accuracy and efficiency are visualized in figure 5*a*. Here, we report the $L_{2}$ error, averaged over all time steps and test trajectories. The single time-scale scheme curves have a ‘U’ shape for each example, indicating that accuracy first improves and then deteriorates as we spend more time in the computation. This finding is consistent with [30], which states that there is a problem-dependent sweet-spot for the hyperparameter Δt. Our multiscale HiTS always achieves the best accuracy, usually with a reasonable computational efficiency, due to the vectorized computations of array programming. For the cubic oscillator, Van der Pol oscillator, and Lorenz system, there seems to exist a single-scale neural network time-stepper with higher efficiency and competitive accuracy compared to our multiscale scheme. This is due to the greedy method we use for the cross validation process: we always prefer models with higher accuracy at the cost of efficiency. However, one can adjust the balance between accuracy and efficiency depending on the objective. The hyperparameter tuning of Δt is implicitly conducted in the cross validation process before coupling the various scales.

**Figure 6.**
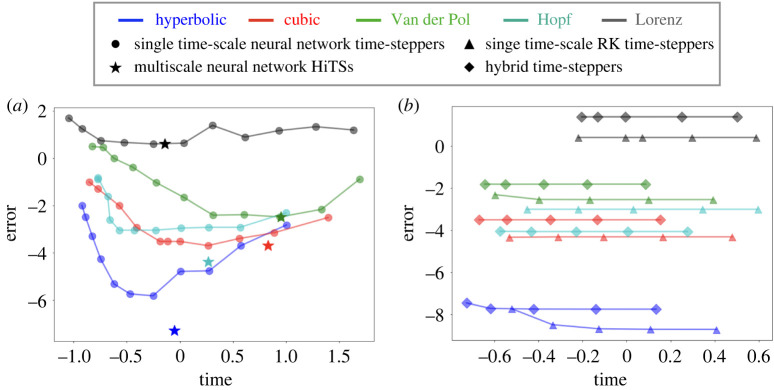
Accuracy versus computational efficiency plot. (*a*) Comparison between multiscale neural network time-stepper and all single time-scale neural network time-steppers. (*b*) Comparison between hybrid time-stepper and Runge–Kutta time-steppers with uniform step sizes. In both plots, horizontal and vertical axes represent time and integrated $L_{2}$ error respectively, visualized in the base-10 logarithmic scale. (Online version in colour.)

Though the multiscale scheme improves the computational efficiency of neural network time-steppers, they still cannot easily match the efficiency of most classic numerical algorithms (see electronic supplementary material, appendix B(c) for more details). Indeed, evaluating a neural network model (forward propagation) typically requires more computational effort than applying a classic discretization scheme that only involves a few evaluations of the known vector field. A hybrid time-stepper, on the other hand, may break this bottleneck by combining large-scale neural network time-steppers with classic numerical time-steppers to make the computations inherently parallelizable. Therefore, we benchmark the hybrid time-steppers against the classic numerical time-steppers in figure 5*b*. In particular, we use a fourth-order Runge–Kutta integrator, with seven different sizes of uniform time step. For simplicity, the hybrid time-steppers are constructed from the same fourth-order Runge–Kutta (RK4) time-steppers with one large-scale neural network time-stepper at the highest level. More details can be found in electronic supplementary material, appendix A(b).

Figure 5*b* shows the trade-off between accuracy and efficiency for our hybrid time-stepper and the classic RK4 scheme. Our hybrid time-stepper offers an efficiency gain over the Runge–Kutta time-stepper with the same minimal step size. This suggests the marginal benefits of enabling vectorized computation are potentially greater than the costs of evaluating a neural network time-stepper. But the accuracy of hybrid time-steppers are usually slightly lower than the purely numerical time-steppers with rare exceptions (e.g. the Hopf normal form example), as the global error is usually dominated by the error of neural network time-steppers, which are model agnostic and purely data-driven, limiting their ability to produce high-fidelity simulations.

### (b) Benchmark on sequence generation

In addition to integrating simple low-dimensional dynamical systems, here we show that it is possible to forecast the state of more complex, high-dimensional dynamical systems. In the field of machine learning, this is often termed *sequence generation*. Importantly, we benchmark our architecture against state-of-the-art networks, including long short-term memory networks (LSTMs) [64], echo state networks (ESNs) [46], and clockwork recurrent neural networks (CW-RNNs). Here, our goal is to train different architectures that can generate the target sequence as accurately as possible. The sequences we explore include a simulated solution of the Kuramoto–Sivashinsky (KS) equation, a music excerpt from Bach’s Fugue No. 1 In C Major, BWV 846, a simulation of fluid flow past a circular cylinder at Reynolds number 100 [81,82], and a video frame of blooming flowers. Within each individual experiment, the various architectures have nearly the same number of parameters; more details about the data preprocessing and choice of parameters are described in electronic supplementary material, appendix A(c).

**Figure 7.**
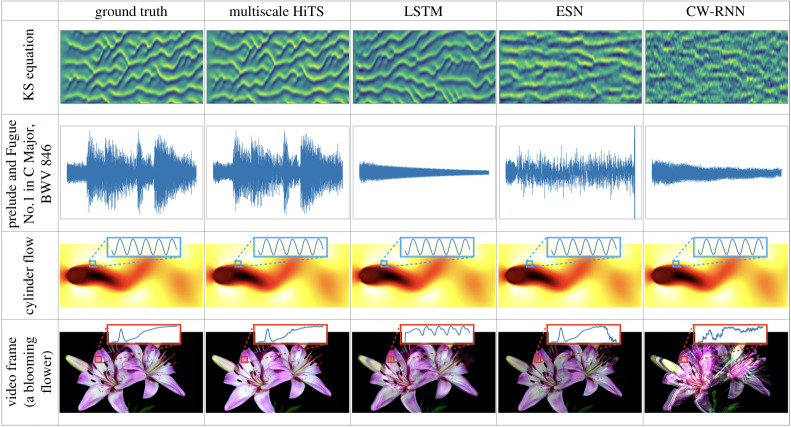
Outputs of different network architectures (column) on each training sequence (row). We use different visualization schemes to show the results: for the KS equation and the music excerpt, we plot the time series evolution, that is, the horizontal axes represent time; for the cylinder flow and the video frame, since each state is a two-dimensional array, we choose to visualize the last frame of our reconstruction, however, we also visualize the time evolution of some states averaged over a small patch of pixel values. For a video that shows the performance, visit: https://youtu.be/2psX5efLhCE. (Online version in colour.)

From figure 7, one can visually see that the multiscale HiTS provides the best sequence generation results, and these results are confirmed by the integrated $L_{2}$ errors shown in table 1. We also see that the LSTM and CW-RNN can learn the first few steps accurately, whereas the ESN tends to smooth the signals. It should be noted that our sequence generation task are very different from the tasks considered in [83], where they use observations of the system’s past evolution to predict future states. An ESN is usually trained by finding a set of output weights through linear regression, if the reservoir is large enough, it can in principle reproduce the observed dynamics perfectly, which would make it an inappropriate benchmark. An ESN with the same number of parameters as the other architectures often cannot fully describe the dynamics, resulting in coarse or smoothed approximations.

**Table 1.** The integrated $L_{2}$ error between the generated sequence and the exact sequence.

sequences	HiTS	LSTM	ESN	CW-RNN
fluid flow	$1.23\times 10^{-7}$	$9.20\times 10^{-8}$	$2.22\times 10^{-8}$	$6.79\times 10^{-7}$
video frame	$7.09\times 10^{-5}$	$1.44\times 10^{-2}$	$1.62\times 10^{-3}$	$8.05\times 10^{-2}$
music data	$8.59\times 10^{-7}$	$4.65\times 10^{-5}$	$2.69\times 10^{-4}$	$4.70\times 10^{-5}$
KS equation	$1.04\times 10^{-3}$	$3.50\times 10^{-0}$	$3.51\times 10^{-0}$	$3.59\times 10^{-0}$

In a nutshell, the competing architectures fail to capture the long-time behaviours, as error accumulation is inevitable for serialized computations. However, our proposed framework should not be viewed as a replacement for these state-of-the-art methods, as they take a different approach and philosophy for sequence generation. RNNs tend to uncover the full dynamics with the help of memory in their internal states, whereas our scheme performs reconstruction using the time-stepping schemes learned at different time scales, though these schemes may only be accurate for a few steps. Instead, our multiscale framework should be used to strengthen these existing approaches, as it can use data-driven models across different scales, avoiding local error accumulations and potentially boosting the accuracy and efficiency.

## 4. Conclusion and discussion

In this work, we have demonstrated an effective and general data-driven time-stepper framework based on synthesizing multiple deep neural networks for hierarchical time-steppers (HiTSs) trained at multiple temporal scales. Our approach outperforms neural networks trained at a single scale, providing an accurate and flexible approach for integrating nonlinear dynamical systems. We have carefully explored this multiscale HiTS approach on several illustrative dynamical systems as well as for a number of challenging high-dimensional problems in sequence generation. In the sequence generation examples, our approach outperforms state-of-the-art neural network architectures, including LSTMs, ESNs and CW-RNNs. Our method explicitly takes advantage of dynamics on different scales by learning flow-maps for those different scales. The coupled model still maintains computational efficiency thanks to the vectorized computations of array programming. Moreover, exactly due to this coupling scheme, each individual network can focus on their intrinsic ranges of interest, bypassing the exploding/vanishing gradient problem for training recurrent neural networks. In addition, we demonstrate the joint use of our neural network time-steppers with the classical time-steppers, resulting in a new computational paradigm: numerical simulation algorithms are now parallelizable rather than serialized in nature, leading to performance boosts in computational speed.

This work highlights fundamental differences between physics-based simulation models and data-driven models. In the former, the errors of the time-stepping constraints are determined strictly by Taylor series expansions which are local in nature and limit the time-step Δt. The latter is a more general flow-map construction that can be trained for any time step Δt. Thus, the error is not limited by a local Taylor expansion, but rather by pairs of training data mapping the solution to a future Δt. In the end, we show our proposed scheme is capable of learning long-term dependencies on some more realistic datasets, achieving state-of-the-art performance.

However, some cautionary remarks are warranted: as with all DNN architectures, obtaining reliable large scaled time-steppers comes at the cost of significant training because of the increasing complexity (see electronic supplementary material, appendix B(d)). Specifically, one usually needs to acquire large enough data sets to train the appropriate deep NN architecture. Deriving a rigorous *a priori* error bound using neural networks is rather involved compared to traditional time-stepping algorithms [84]. Deriving error bounds requires a rigorous formulation of the function space and introduction of an appropriate norm. One remedy for this is to evaluate the trained model on some unseen test trajectories so that the generalization error can be empirically controlled before putting into practical use. Lastly, we have assumed full observation of the system states, although full-state measurements are often unavailable in real-world applications. When the system states are partially observed, techniques such as time-delay embedding [80,85–87] may be applied as a preprocessing step to address the presence of latent variables, which is related to the closure problem.

This work also suggests a number of open questions that motivate further investigation. In particular, nearly every sub-field within numerical integration can be revisited from this perspective. For example, bringing the idea of adaptive step sizes into this framework may potentially lead to even more efficient and accurate time-stepping schemes. Although this important element may be naturally addressed, it is not yet built in to the proposed methodology in its present form. In addition, as mentioned in electronic supplementary material, appendix B(d), the increments of the flow maps mostly exhibit obvious multiscale features. Since deep learning is essentially an interpolation method [88], to leverage more effective training, new sampling strategies may be proposed to address this problem [18,89]. This is of crucial importance for deep learning, as neural networks are data-hungry and the curse of dimensionality makes it impossible to generate largely enough datasets for high-dimensional systems. It is also important to introduce principled approaches to deal with uncertainty quantification within this framework, as real-world systems may be stochastically forced. Similarly, models at different scales may have different associated levels of confidence, which the current multiscale modelling approach does not address. These are both important areas of ongoing research. Bayesian statistical techniques provide one potential approach to enhance the functionality of these methods, by making distribution, rather than point, forecasts.

## Data Availability

All code used to produce the results in this paper is open source at https://github.com/luckystarufo/multiscale_HiTS.
